# Budd–Chiari syndrome caused by latent hepatic metastasis from a thymoma

**DOI:** 10.1016/j.rmcr.2021.101492

**Published:** 2021-08-03

**Authors:** Tomoya Horiguchi, Yoko Toyama, Yosuke Sakakibara, Aki Ikeda, Hisashi Kako, Takuma Ina, Takuya Okamura, Sakurako Uozu, Yasuhiro Goto, Kohei Yokoi, Kazuyoshi Imaizumi

**Affiliations:** Department of Respiratory Medicine, Fujita Health University School of Medicine, Toyoake, Aichi, Japan; Department of Thoracic Surgery, Nagoya University Graduate School of Medicine, Nagoya, Japan; Department of Respiratory Medicine, Fujita Health University School of Medicine, Toyoake, Aichi, Japan

**Keywords:** Thymoma, Liver metastasis, Budd-Chiari syndrome, Ascites, BCS, Budd–Chiari syndrome, CAMP, cisplatin, doxorubicin, and methylprednisolone, IVC, inferior vena cava, SAAG, serum-ascites albumin gradient

## Abstract

A 34-year-old woman visited our hospital because she had had abdominal bloating for 2 months. She had been diagnosed with invasive thymoma (WHO pathological type B2), for which she had undergone chemotherapy and total thymectomy 10 years previously. Six years previously, pleural dissemination was diagnosed and she had undergone right extra-pleural pneumonectomy. On presentation to our hospital, abdominal computed tomography and ultrasound scans revealed abundant ascites and a huge liver lesion, likely a metastasis from her thymoma, obstructing the inferior vena cava. The serum–ascites albumin gradient was high at 1.4 g/dL, which indicated portal hypertension. We diagnosed Budd–Chiari syndrome caused by liver metastasis from a previous thymoma. Steroid therapy resulted in shrinkage of her liver tumor and a marked decrease in her ascites. Although rare, Budd–Chiari syndrome caused by liver metastasis from a thymoma is a possible serious complication of advanced invasive thymoma.

## 1 Introduction

Budd–Chiari syndrome (BCS) is characterized by portal hypertension caused by obstruction or stenosis of the hepatic vein or inferior vena cava (IVC) [1]. Primary BCS is typically caused by thrombosis of the IVC as a result of coagulation abnormalities or membrane-like obstruction. Secondary BCS develops because of extrinsic compression or direct invasion of these veins by primary or metastatic liver cancer [2]. Imaging tests such as abdominal ultrasound are useful for diagnosis. We herein present a case of BCS caused by obstruction of the IVC by a huge liver metastasis from a previously treated thymoma.

## 2 Case presentation

**Fig. 1:**
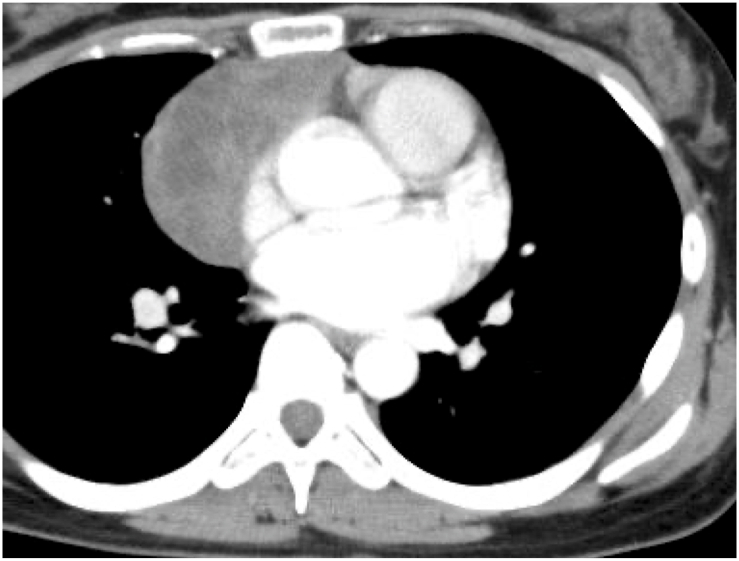
Chest CT image on initial presentation (10 years before the onset of Budd–Chiari syndrome) showing an anterior mediastinal tumor mass with a low-density area. The pathological diagnosis was invasive thymoma (type B2). She underwent systemic chemotherapy (CAMP therapy) followed by total thymectomy at that time. CT, computed tomography.

A 34-year-old non-smoking woman visited our hospital because of worsening abdominal bloating over 2 months. Ten years previously, she was diagnosed with invasive thymoma type B2 (Fig. 1), for which she underwent CAMP therapy (cisplatin, doxorubicin, and methylprednisolone) followed by total thymectomy [3]. Four years previously (6 years after the thymectomy), she was treated with CAMP again, followed by extra-pleural pneumonectomy, for right pleural dissemination. Two years previously (8 years after the thymectomy), she was found to have a recurrence in the right pleural cavity as well as liver metastasis, but then elected to stop attending the hospital because she was concerned about the potential side effects of further treatment.

**Fig. 2:**
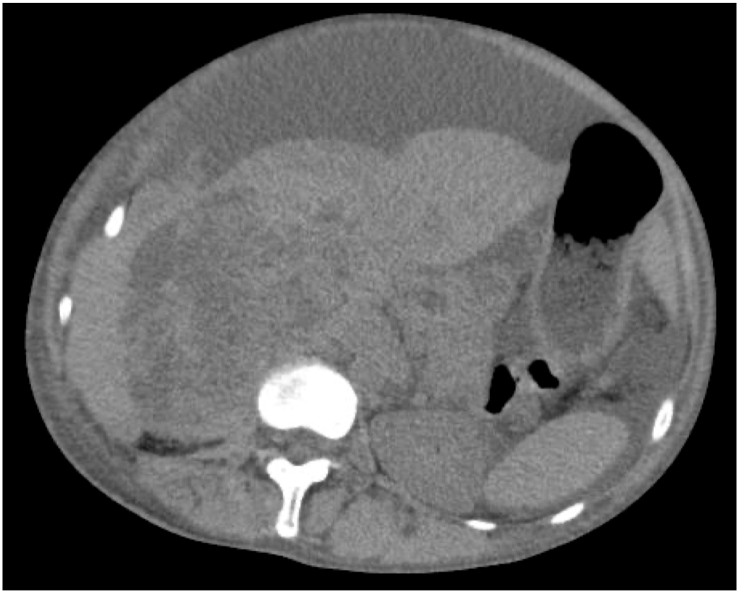
Abdominal CT image on the first visit to our hospital showing a large right hepatic mass of irregular density with indistinct borders. Abundant ascites was also detected.

**Fig. 3:**
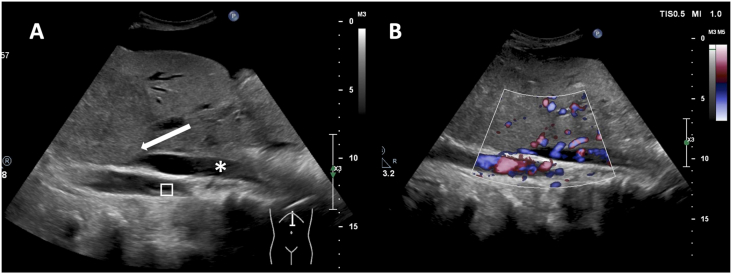
Abdominal ultrasonography findings. (A) A large mass is compressing the inferior vena cava (white arrow). * inferior vena cava, abdominal aorta. (B) A color doppler image showed congestion of blood flow in the inferior vena cava. . (For interpretation of the references to color in this figure legend, the reader is referred to the Web version of this article.)

Physical examination on admission revealed marked abdominal distension and bilateral leg edema. She did not show caput medusae nor splenomegaly. Her performance status on admission was Eastern Cooperative Oncology Group (ECOG) grade 3. Laboratory studies revealed a white blood cell count of 14,400/μL, 38.5% hematocrit, a platelet count of 269,000/μL, mild hepatic impairment (total bilirubin 1.1 mg/dL, aspartate aminotransferase 35 IU/L, alanine aminotransferase 20 IU/L, lactate dehydrogenase 342 IU/L, alkaline phosphatase 574 IUL/L, gamma-glutamyl transpeptidase 63 IU/L, leucyl aminopeptidase 107 IU/L), and normal renal function (creatinine 0.63mg/dL, estimated glomerular filtration rate 86.4 mL/min/1.73 m^2^). An abdominal ultrasound and a non-enhanced computed tomography scan of the chest and abdomen showed a large amount of ascites and massive lesions with irregular borders in the S2 segment of the liver, peritoneum, and right pleural cavity (Fig. 2). Ascites cytology was negative for malignancy. A culture of the ascitic fluid was also negative. The ascites albumin concentration was 1.9 g/dL and the serum–ascites albumin gradient (SAAG) was 1.4 g/dL (>1.1 g/dL), which suggested portal hypertension. An abdominal ultrasound clearly showed marked compression of the IVC and blood flow limitation by a large tumor (Fig. 3). She had no thymoma-associated paraneoplastic disease such as myasthenia gravis, pure red cell aplasia, nor agammaglobulinemia.

Taking these findings together, we diagnosed BCS caused by a huge thymoma metastasis in the liver. We performed ascites drainage twice (1600 ml each) during her hospitalization, but her symptoms of abdominal bloating did not improve. Administration of steroid pulse therapy (methylprednisolone 1 g for 3 days) followed by prednisolone 25 mg for 7 days resulted in significant symptomatic improvement, and abdominal CT showed shrinkage of the liver metastasis and a reduction in the ascites. Her body weight (70 kg before commencing steroid treatment) fell to 60.8 kg after two cycles of steroid pulse therapy. She was discharged from the hospital on tapering doses of steroids.

## 3 Discussion

Secondary BCS is mainly caused by tumor invasion accompanied by compression of the IVC and/or hepatic vein [4]. Many abdominal tumors, including primary liver cancer and metastatic renal cancer, can cause BCS. However, there have only been a few reports of BCS caused by liver metastases from thoracic tumors, including lung cancer [2]. In particular, to the best of our knowledge, there are no published reports of secondary BCS caused by thymoma metastasis in the liver. Because the patient refused to receive any invasive procedures including liver biopsy, we could not obtain pathological confirmation of the liver mass. Although our initial differential diagnosis discussion included hepatoma or lymphoma, we concluded from her medical history of repeated recurrence of thymoma that the liver mass was metastasis of invasive thymoma.

Thymic tumors, especially thymic carcinoma and high-risk thymoma (B2, B3 WHO classification), sometimes metastasize to distant sites post-operatively. The pleura is the most common site of thymoma metastasis, followed by the thoracic lymph nodes [5]. Extra-thoracic metastases of thymic tumors are rare; the liver is the second most common extra-thoracic metastatic site [6]. Notably, thymoma relapses can be diagnosed years after resection of the primary tumor [7]. In our case, liver metastasis was first detected 8 years after thymectomy. Thus, latent metastases from thymomas can present in unexpected ways.

We initially suspected that our patient's ascites was attributable to peritoneal dissemination of the thymoma. However, her high SAAG (1.4 g/dL) led us to suspect portal hypertension, which in turn led to the diagnosis of BCS, this being confirmed by imaging. A high SAAG (>1.1 g/dL) is a useful indicator of portal hypertension [8], and was critical to the diagnosis in this case.

We discussed several options for reducing our patient's IVC obstruction. The possibilities included chemotherapy, interventional radiology, radiation therapy, or resection of the liver tumor. Because her liver metastasis was large and she also had thymoma metastases in the thorax, her general condition was poor. We therefore considered that systemic chemotherapy, radiation, or surgery were inadvisable. Given that steroid monotherapy can reportedly be effective against metastatic thymoma [9], we initiated steroid pulse therapy. This resulted in a remarkable decrease in the ascites and a moderate reduction in the liver tumor diameter, from 78.9 mm to 60.6 mm. We speculate that release of IVC decompression caused by tumor shrinkage resulted in an improvement in the venous return and renal fluid clearance.

## 4 Conclusions

Invasive thymoma is sometimes refractory to multimodal therapy. Rarely, latent metastases from thymomas cause serious complications such as BCS.

## Funding

This research did not receive any specific grant from funding agencies in the public, commercial, or not-for-profit sectors.

## Declaration of competing interest

The authors have no conflicts of interest or financial ties to disclose with respect to publication of this article.
